# Effects of integrated application of plant-based compost and urea on soil food web, soil properties, and yield and quality of a processing carrot cultivar

**DOI:** 10.21307/jofnem-2020-111

**Published:** 2020-10-21

**Authors:** A. Habteweld, D. Brainard, A. Kravchencko, P. S. Grewal, H. Melakeberhan

**Affiliations:** 1 Department of Horticulture, Michigan State University, East Lansing, MI, 48824; 2 Department of Plant, Soil and Microbial Sciences, Michigan State University, East Lansing, MI, 48824; 3 College of Sciences, University of Texas Rio Grande Valley, Edinburg, TX, 78539; 4 Department of Entomology and Nematology, University of Florida, Gainesville, FL, 32611-0620

**Keywords:** Compost, Ecology, Integrated application, Nematodes, Soil health, Trophic group

## Abstract

Soil nutrient management system characterized by reduced input of inorganic fertilizers integrated with organic amendments is one of the alternatives for reducing deleterious environmental impact of synthetic fertilizers, suppressing soil-borne pests and diseases, and improving soil health and crop yield. A hypothesis of the present study was that lower rates of urea mixed with higher rates of plant compost (PC) would improve nematode community structure, soil food web condition, soil biological, and physiochemical properties, and yield and quality of a processing carrot (*Daucus carota*) cultivar. Urea and PC were each applied at 135 kg nitrogen (N)/ha alone or at 3:1, 1:1, and 1:3 ratios annually during the 2012 to 2014 growing seasons. A non-amended check served as a control. Nematode community was analyzed from soil samples collected approximately 4-week intervals from planting to 133 days after planting each year. Soil respiration, as a measure of soil biological activity, and soil physiochemical properties were determined from soils collected at planting and at harvest in 2012 and 2013. Results showed that PC alone, and U1:PC1 resulted in soil food web structure significantly above 50 at harvest in 2014. Urea significantly decreased end-of-season soil pH, but increased NO_3_-N compared with the other treatments. While the herbivore population density was low, abundances of *Tylenchus* and *Malenchus* were negatively correlated with carrot fresh weight of marketable carrot. Overall, results suggest that integrating lower rates of urea and higher rates of PC are likely to increase soil biological activity, soil pH, and phosphorus content.

Maintaining soil and water quality, and obtaining optimum crop yields are major components of sustainable agriculture (Doran, 2002; Evanylo et al., 2008). Excessive amount of inorganic fertilizers, nitrogen (N) in particular, are applied and replenished in every growing season in order to achieve a high crop yield and satisfy the demand of an increasing human population (Anwar et al., 2005; Stewart et al., 2005). These fertilizers are rapidly lost and pose deleterious effects to the environment and human health (Tilman et al., 2002; Gruzdeva et al., 2007). The sole use of inorganic fertilizers is also causing deterioration in soil physical, chemical, and biological properties (Odunze et al., 2012; Eche et al., 2013; Singh et al., 2013).

In contrast, organic amendments increase availability of nutrients, improve soil structure leading to better moisture retention and soil microbial activity and reduce fertilizer loses to the environment (Evanylo et al., 2008; Mylavaropu and Zinati, 2009; Natsheh and Mousa, 2014; Zhang et al., 2014, 2016). Such positive traits increase agricultural productivity with minimum damage to the environment (Oquist et al., 2007; Forge and Kempler, 2009; Glover et al., 2010). However, use of compost amendments alone is usually not sufficient to maintain the expected productivity level as that of synthetic fertilizers in the short-term (Pimentel et al., 2005; Herencia et al., 2008; Diacono and Montemurro, 2010). Integrated plant nutrient management system characterized by reduced input of inorganic fertilizers integrated with organic amendments is one of the alternatives to achieve the expected yield while reducing the deleterious environmental impact of synthetic fertilizers (Oberson et al., 1996; Gunapala and Scow, 1998; Kaur et al., 2005; Pimentel et al., 2005). Hence, nutrient management is a key entry point for sustainable agricultural productivity (Chivenge et al., 2009).

Combination of readily available inorganic fertilizers can solve soil nutrient deficits while mineralization of the organic component improves soil biological and physiochemical properties, and enhance yield over time (Noor et al., 2007; Odunze et al., 2012; Eche et al., 2013). Moreover, integrated application of nitrogen fertilizers with compost improved nitrogen utilization efficiency of plants (Keeling et al., 2003; Ahmad et al., 2006). Thus, integrated application of inorganic fertilizers and compost that utilizes compost at lower than fertilizer rates and reduces the amount of inorganic fertilizers applied to soil and the accumulation of non-nutrient constituents such as heavy metals is an appealing strategy (Sikora and Knkiri, 2001). Implementation of such an alternative could be best achieved if its effects on soil food web, which drives nutrient transformations and productivity, are better understood.

As the most abundant organisms in the terrestrial ecosystems and occurring at multiple levels of the soil food web, nematodes are key drivers of the soil food web (Yeates et al., 1993) and provide insights of the soil conditions (Bongers and Bongers, 1998; Ferris et al., 2001). Nematodes are also considered as a powerful indictor of soil ecosystem change (Freckman and Ettema, 1993; Wasilewska, 1994; Ferris and Matute, 2003; Ferris et al., 2004; Cheng and Grewal, 2009; Knight et al., 2013). The soil food web structure and function was graphically described as a function of EI (measure of opportunistic bacterivores and fungivores in the community) and SI (indicator of food web status affected by stress or disturbance) as described by Ferris et al. (2001).

While there are several studies on the impact of organic amendments and inorganic fertilizer on nematode community, soil fertility, and plant productivity (Bulluck et al., 2002a; Briar et al., 2007), the impact of mixed compost-fertilizer applications on soil nematodes community structure and overall soil food web health is less understood. The objectives of this study were to compare the effects of mixed application of different levels of plant leaf-based compost (PC) and urea on nematode community structure, soil food web condition, soil biological and physiochemical properties, and yield and quality of a processing carrot cultivar ‘Cupar’ in a sandy loam soil. The central hypothesis is that lower rates of urea mixed with higher rates of PC would improve nematode community structure, soil food web condition, soil biological and physiochemical properties, and increase carrot yield and quality relative to single applications of either product.

## Material and methods

### Experimental design and treatments

A field experiment with a randomized complete block design with four replications was conducted at Michigan State University (MSU) Horticulture Teaching and Research Center in Holt, Michigan (N 42°40.326′, W 084°28.922′, 847 m elevated) in a Marlette fine sandy loam (fine-loamy, mixed, mesic Glossoboric Hapludalfs, Anonymous, 1977) during 2012 to 2014 growing seasons. Prior to the start of the experiment carrots were grown in the field and amended with composted manure in 2010 and 2011 growing seasons. The experiment had a total of 24 plots (6 treatments × 4 replications). Each plot was 3.72 meter square (3.05 m × 1.22 m) and had four rows. The plots were separated by 1.83 m wide guard rows between their length and 1.52 m between their widths. PC from leaves of different plant species left to decompose for more than 10 years obtained from MSU Student Organic Farm, Holt, MI, USA, analyzed for nutrient contents, and applied on dry matter basis (Habteweld et al., 2018). The standard urea was obtained from MSU Horticulture Teaching and Research Center in Holt, Michigan. The recommended rate of urea for processing carrots is 135 kg N/ha. Standard urea and PC were mixed and applied at 1:0 (U1:PC0), 3:1 (U3:PC1), 1:1 (U1:PC1), 1:3 (U1:PC3), and 0:1 (U0:PC1) ratio each year before planting to provide 135 kg N/ha. Non-amended check served as control. The field was tilled to the depth of 30 cm and treatments per plot were uniformly applied by hand at planting and mixed to 10 cm soil depth using an RTR2548 rototiller (Land Pride).

### Soil sampling, and nematode extraction, identification, and enumeration

Approximately 500 g of soil from a composite six soil cores collected from center two rows of each plot at 0, 32, 62, 94, and 133 days after planting in 2012, 2013, and 2014 growing seasons. A total of 15 soil samplings (3 years × 5 sampling dates per year) were performed from each plot. The composite soil samples were stored in a cold room at 5°C for 3 to 5 days before the nematodes were extracted from 100 cc of fresh soil using a semi-automatic elutriator (Avendaño et al., 2003). Nematodes were fixed in double TAFF (Hooper, 1986), identified and enumerated at genus level using an inverted microscope (Accu-scope Inc, Commack, NY, USA) at 400 × magnification following diagnostic keys by Bongers (1994) and the University of Nebraska Lincoln nematode identification website (http:nematode.unl.edu/konzlistbutt.htm). Nematodes were then assigned to herbivore, bacterivore, fungivore, omnivore, or predator trophic group (Yeates et al., 1993; Okada and Kadota, 2003) and a colonizer persister (c-p) groups (Bongers and Bongers, 1998).

### Nematode community analysis

Shannon–Weaver diversity index [H′ = *−*Σ*pi* (ln *Pi*)], where *Pi* is the proportion of genus taxa in the nematode community *n* (Shannon and Weaver, 1949), Hill’s diversity N1 [exp(H′)] and N0 (genera richness = number of all genera in the same community) (Hill, 1973) were calculated. Nematode community maturity indices such as maturity index (MI) (includes c-p 1 to c-p 5 non-herbivores), MI25 (includes only c-p 2 to c-p 5 non-herbivores) and plant-parasitic index (PPI) (includes c-p 2-5 herbivores) were calculated according to Bongers (1990). These indices were calculated as weighted mean frequency, mathematically expressed as Σ(*vi* × *fi*)/*n* where *vi* is c-p value assigned to nematode genus *i*; and *fi* is the frequency of nematode genus *i* and *n* is total number of individuals in a sample (Bongers, 1990).

### Soil food web analysis

Basal (BI), EI, and SI and channel (CI) indices were calculated according to Ferris et al. (2001) based on the weighted abundance of nematode guilds representing structure (*s* = Σ*k*_*s*_*n*_*s*_), enrichment (*e* = Σ*k*_*e*_*n*_*e*_), and basal (*b* = Σ*k*_*b*_*n*_*b*_), where *k* is the specific weight of each guild and *n* is the relative frequency of each nematode functional guild in the soil sample using the following formulas: BI = 100[*b*/(*e* + *s* + *b*)], SI = 100[*s*/(*s* + *b*)] and EI = 100[*e*/(*e* + *b*)] was calculated based on the ratio of fungivores of c-p 2 with the decomposer guilds (fungivores of c-p 2 and bacterivores of c-p 1) as 100[0.8 (fungivores of c-p 2)/[3.2 (bacterivores of c-p 1) + 0.8 (fungivores of c-p 2)]. Nematode faunal profile was graphically described as function of EI (indicator nutrient availability) and SI (indicator of food web food web complexity).

### Soil physiochemical properties

Changes in soil pH, macro and micronutrients, percent of soil moisture content, and bulk density, and soil respiration were measured before planting and at harvest in 2012 and 2013 growing seasons. Soil moisture content level in each sample was determined by weight loss after oven dry at 104°C for 24 hr. Bulk density measurements were done drying cores of soil at 104°C for 24 h (Blake and Hartge, 1986). Soil pH, phosphorus, potassium, calcium, magnesium, soil organic matter, nitrate-nitrogen, ammonium-nitrogen, and cation exchange capacity were determined by the MSU Soil and Plant Nutrient Laboratory using standard procedures (Huffman and Barbarick, 1981; Nelson, 1983). The rate of CO_2_ emission from the soil samples was used as an indicator of relative soil respiration and of level of biological activity (Ettema et al., 1998; Ferris and Matute, 2003; Treonis et al., 2010). In total, 15 g of fresh soil sample was incubated in 237 ml glass jars at 22°C for 7 days at field soil moisture content during sampling (Treonis et al., 2010). The CO_2_ concentration of a 0.5 ml headspace gas sample was withdrawn from the jar through the rubber stopper using 1 ml syringe. The concentration was determined after 7 days of incubation using an infrared gas analyzer (LI-820, LI-COR, Inc., Lincoln, NE, USA; Zibilske, 1994) and expressed as µg CO_2_-C per gram of soil per day.

### Carrot yield and quality

Carrots were harvested from the center two rows using spading fork (True Temper, AMES companies, Inc.) and washed with tap water using a garden hose. Carrots were categorized as marketable, when the length was greater than or equal to 13 cm and the diameter at the shoulder was greater than 2.5 cm without defects, and unmarketable, when they were stunted, less than 2.5 cm with cracks, forks, and rotting defects (Anonymous, 1965).

### Data analysis

Nematode taxa and trophic group abundances were expressed on an absolute basis (number of nematodes in a taxon *i* per 100 cc of fresh soil). Nematode taxa and trophic group abundances were not expressed per 100 g of dry soil because soil moisture content was not measured during all sampling times except before planting and harvest. Because of that the nematode taxa and trophic group abundances could not be converted in dry soil basis for all sampling times. Nematode abundance data were transformed as ln (*x* + 1) prior to statistical analysis to normalize variance. Treatments were compared for nematode trophic groups, community indices, soil food web indices, soil, and yield variables. Statistical analysis was conducted using the PROC MIXED procedure of SAS. The statistical model consisted of fixed effects of amendments and sampling time, and the interaction between them, and random effects of blocks and block by amendment interaction. The interaction between blocks and amendments was used as an error term to test the effect of amendments. The effect of time was addressed using the repeated measures approach with REPEATED statement of the PROC MIXED. Akaike information criterion was used to select the optimal variance-covariance structure for the repeated measures analysis.

**Figure 1:**
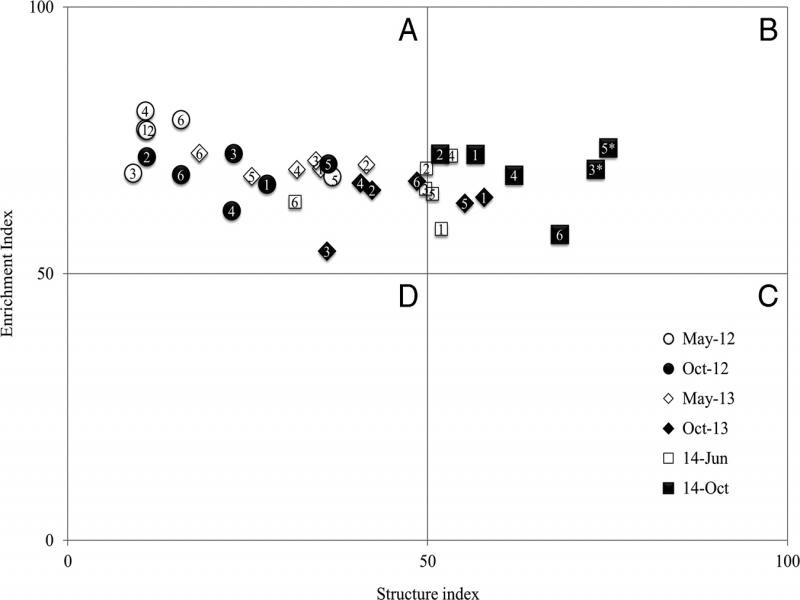
Soil food web condition in plots amended with integrated application of urea and PC, standard urea and non-amended check in sandy loam soil at planting (May, June) and harvest (October) in 2012 to 2014 growing seasons. Numbers 1 to 6 represent treatments: 1 = Urea alone (U1:PC0), 2 = U3:PC1, 3 = U1:PC1, 4 = U1:PC3, 5 = PC alone (U0:PC1), and 6 = non-amended check. The soil food web condition is expressed in four quadrants (A, B, C, and D) according to Ferris et al. (2001). *Treatments significantly increased SI from 50 using one-tail *t*-test at *α* = 0.05 for 2014 growing season.

In order to further evaluate changes in soil food web condition, SI analyzed in three ways. First, the SI values were compared following a standard ANOVA and mean separations as part of the food web parameters. Second, SI values were plotted to the food web model (Fig. 1). Third, SI values were tested for statistical difference from 50 (cut off point of the soil food web structure) using one-tail *t*-test at *α* = 0.05. The means of the treatments with statistical difference from 50 are noted by asterisks (*).

Yield parameters were compared using one-way analysis of variance (PROC MIXED, SAS ver 9.3, SAS Institute Inc., 2012, Cary, NC, USA). The statistical model consisted of fixed effect of amendments and a random effect of block and block by amendment. The interaction between blocks and amendments was used as an error term to test the effect of amendments. Interaction effects of amendment and sampling time are presented in results only when they were significant. Otherwise, we have presented only significant main effects of treatment. The probability level *P* ≤ 0.05 was regarded as significant.

The relationships among nematode trophic groups and soil physiochemical properties, and soil food web indices, soil physiochemical properties and yield parameters were separately analyzed in multiple factor analysis (MFA) (Escofier and Pages, 1994) using R-program (R v. 4. 0. 0). MFA analysis helps to get the best linear combinations of the original variables on Dimension 1 and 2, which represent the first and second best summary of variability of the information, respectively. Variables with positive correlations to dimension 1 and 2 are related to each other. The variables closer to −1 on each axis are negatively correlated to variables closer to 1.

## Results

### Effect on nematode community structure

A total of 51 nematode genera were identified in the plots throughout the study period (Table 1). The number of genera identified as herbivores, bacterivores, fungivores, omnivores, and predators were 17, 16, 6, 7, and 5, respectively. Among herbivore nematodes, *Malenchus, Pratylenchus, Helicotylenchus*, and *Tylenchus* were the most abundant genera and represented 11, 11, 24, and 36%, respectively. *Mesorhabditis, Microlaimus, Acrobeloides*, and *Rhabditis* represented 10, 10, 21, and 22% of the bacterivores, respectively. *Filenchus and Aphelenchus* represented 33 and 46% of fungivores, respectively. The abundances of omnivores and predators were generally low representing less than 3% of the total nematode community. Nematode trophic group abundance, and nematode maturity (MI, MI25, and PPI) and diversity (H′, Hill’s N0 and N1) indices were significantly affected by sampling time, but not by the interaction of treatment and sampling time, or treatment (Table 2).

**Table 1.** List of nematode genera detected in plots amended with integrated application of urea and PC at different levels to supply 135 kg N/ha recommended for processing carrot cultivars, standard urea, and non-amended check plots in sandy loam soil in 2012, 2013, and 2014 growing seasons.

Herbivores	Bacterivores	Fungivores	Omnivores	Predators
*Basiria (2)*	*Eumonhystera (1)*	*Aphelenchoides (2)*	*Eudorylaimus (4)*	*Tripyla (3)*
*Boleodorus (2)*	*Mesorhabditis (1)*	*Aphelenchus (2)*	*Mesodorylaimus (4)*	*Clarkus (4)*
*Cephalenchus (2)*	*Panagrellus (1)*	*Ditylenchus (2)*	*Microdorylaimus (4)*	*Mylonchulus (4)*
*Malenchus (2)*	*Panagrolaimus (1)*	*Filenchus (2)*	*Pungentus (4)*	*Prionchulus (4)*
*Paratylenchus (2)*	*Pellioditis (1)*	*Diphtherophora (3)*	*Thonus (4)*	*Nygolaimus (5)*
*Psilenchus (2)*	*Pristionchus (1)*	*Tylencholaimellus (4)*	*Aporcelaimellus (5)*	
*Tylenchus (2)*	*Rhabditis (1)*		*Prodorylaimus (5)*	
*Dolichorynchus (3)*	*Acrobeloides (2)*			
*Helicotylenchus (3)*	*Cephalobus (2)*			
*Hemicycliophora (3)*	*Cervidellus (2)*			
*Heterodera (J2)*^a^ *(3)*	*Eucephalobus (2)*			
*Pratylenchus (3)*	*Heterocephalobus (2)*			
*Rotylenchus (3)*	*Plectus (2)*			
*Tylenchorhynchus (3)*	*Microlaimus (3)*			
*Trichodorus (4)*	*Prismatolaimus (3)*			
*Longidorus (5)*	*Alaimus (4)*			
*Xiphinema (5)*				

**Notes:** Numbers within brackets represent c-p values following Bongers and Bongers (1998). ^a^J2 = Stage 2 juvenile.

**Table 2.** Probability values (Pr > *F*) of treatment (TR), sampling time (T), and interaction of treatment and sampling time (TR × T) effects for nematode trophic group abundances, non-herbivore and total nematodes, nematode community and soil food web indices, soil respiration and soil physiochemical properties for field plots amended with integrated application of urea and PC at different levels to supply 135 kg N/ha and standard urea application and non-amended check in sandy loam soil in 2012 to 2014.

	Probability > *F*		
Variables	TR	*T*	TR × *T*
Trophic groups			
Herbivores	0.95	< 0.0001	0.60
Bacterivores	0.95	< 0.0001	0.67
Fungivores	0.93	< 0.0001	0.77
Omnivores	0.86	< 0.0001	0.35
Predators	0.48	< 0.0001	0.82
Non-herbivores	0.98	< 0.0001	0.62
Total nematodes	0.97	< 0.0001	0.89
Diversity indices			
H′a	0.99	< 0.0001	0.2
Hill’s N1	0.97	< 0.0001	0.29
Hill’s N0	0.99	< 0.0001	0.44
Ecological disturbance indices			
PPI	0.91	< 0.0001	0.43
MI	0.67	< 0.0001	0.48
MI25	0.13	< 0.0001	0.64
Food web indices			
EI	0.630	0.0012	0.49
SI	0.041	< 0.0001	0.48
BI	0.623	< 0.0001	0.61
CI	0.940	< 0.0001	0.48
Soil respiration	0.020	< 0.0001	0.53
Soil physiochemical properties			
Bulk density	0.45	< 0.0001	0.98
Porosity	0.26	< 0.0001	0.98
Moisture	0.28	< 0.0001	0.73
Soil pH	0.18	< 0.0001	0.01
Phosphorus	0.02	0.005	0.74
Potassium	0.69	< 0.0001	0.42
Calcium	0.48	< 0.0001	0.03
Magnesium	0.74	< 0.0001	0.55
Organic matter	0.23	< 0.0001	0.54
Nitrate-nitrogen	0.02	< 0.0001	<0.0001
Ammonium-nitrogen	0.43	< 0.0001	0.99
Cation exchange capacity	0.62	< 0.0001	0.55

**Note:** ^a^Shannon–Weaver diversity index (Shannon and Weaver, 1949).

### Effect on soil food web condition

Soil food web indices (BI, EI, SI, and CI) were not affected by the interaction of treatment and sampling time. However, all the soil food web indices were affected by sampling time (Table 2). The nematode faunal profiles are presented from soil samples collected before planting and at harvest annually (Fig. 1). Except urea in 2013, all the treatments resulted in data falling in Quadrant A (poorly structured soil food webs) at planting in all years. Similarly, all the treatments resulted in data falling in Quadrant A at harvest in 2013. At harvest in 2013, urea and PC resulted in data falling in Quadrant B, enriched and maturing food web. All the treatments had maturing and enriched food webs at harvest in 2014, but only PC alone (*t*(15) = 2.55, *α* = 0.05), and U1:PC1 (*t*(15) = 2.37, *α* = 0.05) showed significantly greater than 50 food web structure (Fig. 1).

### Effect on soil physiochemical properties

Soil pH, calcium, and NO_3_-N were significantly affected by the interaction of treatment and time while soil respiration and other soil physiochemical properties were not affected (Tables 2 and 3). Soil respiration and NO_3_-N was significantly affected by treatment while soil respiration and all the soil physiochemical properties were affected by sampling time.

**Table 3.** Soil pH, nitrate-nitrogen (NO_3_-N) and calcium (Ca) contents (ppm) in plots amended with integrated application of urea and PC to supply 135 kg N/ha recommended for processing carrot cultivars, standard urea application and non-amended check in sandy loam soil at planting (0) and at 133 days after planting (DAP) in 2012 to 2013 growing seasons.

			Treatments as a ratio of urea (U) and PC					
Variables	YR	DAP	U1: PC0^a^	U3:PC1	U1:PC1	U1:PC3	U0:PC1	Check
pH	2012	0	6.8 ± 0.1 bB^b^	6.8 ± 0.2 bB	7.2 ± 0.3 bA	7.0 ± 0.2 bAB	7.2 ± 0.1 bA	7.2 ± 0.4 cA
		133	6.4 ± 0.3 cB	6.8 ± 0.3 bAB	7.4 ± 0.1 abA	7.2 ± 0.4 bA	7.3 ± 0.5 bA	7.3 ± 0.6 abcA
	2013	0	6.9 ± 0.1 bA	7.0 ± 0.2 abA	7.4 ± 0.1 abA	7.2 ± 0.3 bA	7.3 ± 0.5 bA	7.4 ± 0.6 bA
		133	6.7 ± 0.2 bB	7.1 ± 0.1 aAB	7.5 ± 0.1 aA	7.4 ± 0.2 aA	7.6 ± 0.3 aA	7.5 ± 0.5 abA
NO_3_-N	2012	0	1.4 ± 0.3 dAB	0.6 ± 0.2 cB	0.8 ± 0.9 cAB	0.5 ± 0.1 cB	0.7 ± 0.5 cB	1.5 ± 0.6 cA
		133	29.2 ± 17 aA	2.9 ± 0.9 bB	3.0 ± 1 bB	3.7 ± 1 abB	4.6 ± 2 bB	3.0 ± 2 bB
	2013	0	5.3 ± 0.6 cA	5.4 ± 1.3 aA	6.0 ± 0.9 aA	5.4 ± 0.2 aA	6.5 ± 1.2 aA	7.2 ± 3 aA
		133	17.5 ± 8 bA	2.6 ± 1.2 bB	3.4 ± 2.3 bB	4.6 ± 2.5 aB	3.8 ± 1.4 bB	2.7 ± 0.5 bB
Ca	2012	0	1159.7 ± 171 bA	1120.7 ± 123 bA	1263.3 ± 129 abA	1139.3 ± 82 bA	1192.7 ± 115 bA	1295.7 ± 298 bA
		133	1057.3 ± 103 bA	1126.7 ± 110 bA	1287.7 ± 122 abA	1193.7 ± 33 bA	1307.3 ± 351 abA	1303.7 ± 433 bA
	2013	0	1111.3 ± 91 bA	1108.7 ± 120 bA	1246.7 ± 73 bA	1152.3 ± 129 bA	1345.3 ± 424 abA	1406.3 ± 495 aA
		133	1243.3 ± 95 aA	1242.3 ± 121 aA	1471.3 ± 57 aA	1388.3 ± 116 aA	1423.0 ± 186 aA	1450.3 ± 365 aA

**Notes:** ^a^Treatments expressed as urea-to-PC ratio; ^b^means with different lower case letters in columns within each soil variable and different upper case letters across rows indicate the significant difference at *P* ≤ 0.05 using Fisher’s LSD.

Urea significantly decreased soil pH at harvest in 2012 compared with at planting in 2012, but such effect was not observed between samples collected at planting and at harvest in 2013. U1:PC3 and PC significantly increased soil pH at harvest in 2013 compared with the other sampling times. Soil pH in urea, and in U3:PC1 was significantly lower compared with all the other treatments including non-amended check at planting in 2012. However, only urea significantly decreased soil pH at harvest compared with all the treatments in 2012. There was no significant difference in soil pH among the treatments at planting in 2013, except urea significantly decreased soil pH at harvest compared with all the other treatments (Table 3).

All the treatments significantly increased soil NO_3_-N content at harvest compared with at planting in 2012 (Table 3). In 2013, urea significantly increased NO_3_-N at harvest compared with at planting. The integrated treatments, except U1:PC3, and non-amended check, significantly decreased NO_3_-N at harvest than that at planting in 2013. Non-amended check had significantly higher NO_3_-N compared with all the treatments except urea at planting in 2012. Urea significantly increased soil NO_3_-N content compared with all the treatments at harvests in 2012 and 2013.

**Figure 2:**
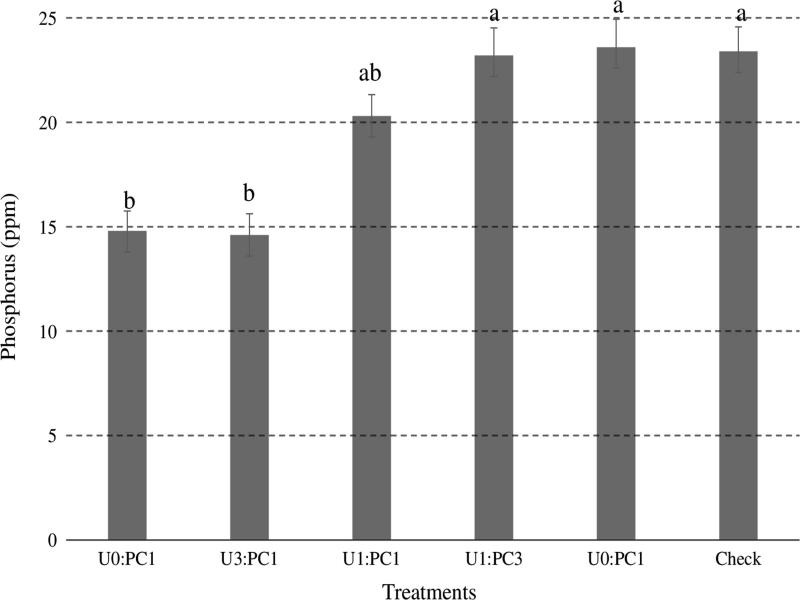
Means across all sampling time points of soil phosphorus content at the studied treatments across 2012 and 2013 growing seasons. Ratios represent treatments: Urea alone (U1:PC0), 3:1 ratio of urea and PC (U3:PC1), 1:1 ratio of urea and PC (U1:PC1), 1:3 ratio of urea and PC (U1:PC3), PC alone (U0:PC1) and Check = non-amended control. Bars with different letters are significantly different at *P* ≤ 0.05 using Fisher’s LSD. Error bars represent standard errors.

**Figure 3:**
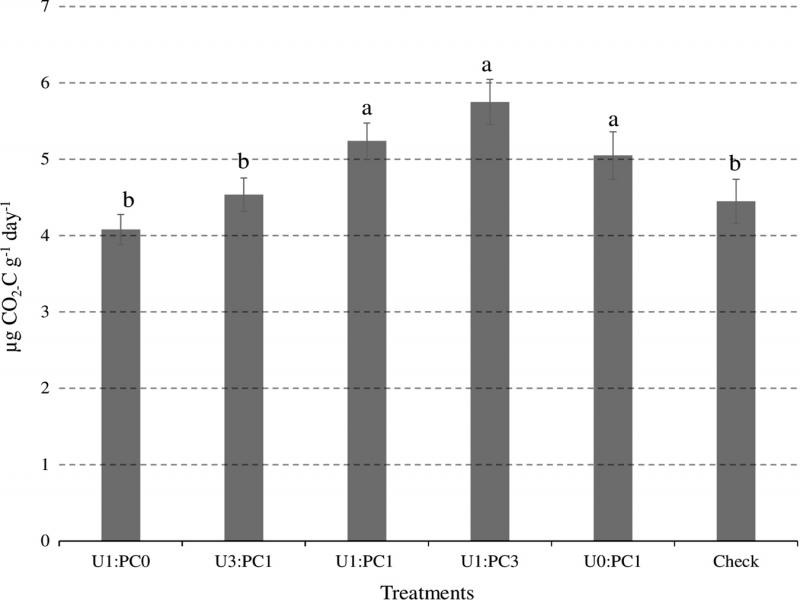
Means across all sampling time points of soil respiration (µg CO_2-_C g^−1^ day^−1^) at the studied treatments across 2012 and 2013 growing seasons. Ratios represent treatments: Urea alone (U1:PC0), 3:1 ratio of urea and PC (U3:PC1), 1:1 ratio of urea and PC (U1:PC1), 1:3 ratio of urea and PC (U1:PC3), PC alone (U0:PC1) and Check = non-amended control. Bars with different letters are significantly different at *P* ≤ 0.05 using Fisher’s LSD. Error bars represent standard errors.

There was no treatment effect on soil calcium content at harvest in 2012. All treatments, except PC and non-amended check, significantly increased soil calcium content at harvest in 2013 compared with at planting in 2013. Urea and U3:PC1 significantly increased soil calcium content at harvest in 2013 compared with at planting and at harvest in 2012, and at planting in 2013. There was no significant difference in soil calcium content among the treatments in all the sampling dates. Urea, U3:PC1 significantly decreased soil phosphorus content compared with all the treatments (Fig. 2). PC, U1:PC3, and U1:PC1 significantly increased soil respiration compared with the rest of the treatments (Fig. 3).

### Effect on carrot yield and quality

**Figure 4:**
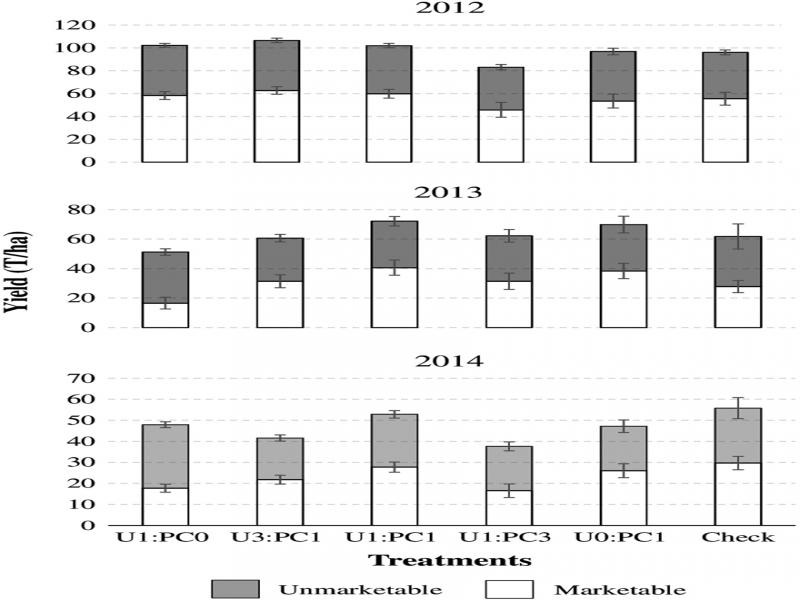
Effect of treatments on mean carrot yield by category (marketable and unmarketable) in 2012, 2013, and 2014. Ratios represent treatments: Urea alone (U1:PC0), 3:1 ratio of urea and PC (U3:PC1), 1:1 ratio of urea and PC (U1:PC1), 1:3 ratio of urea and PC (U1:PC3), PC alone (U0:PC1) and Check = non-amended control. There was no significant difference in quality category at *P* ≤ 0.05 using Fisher’s LSD.

Carrot quantity and quality were not affected by the treatments in any year due to high variability in carrot yield, making it difficult to come to any conclusion about effects on carrot yield (Fig. 4).

### Effect on correlations among nematode, soil, and yield parameters

**Figure 5:**
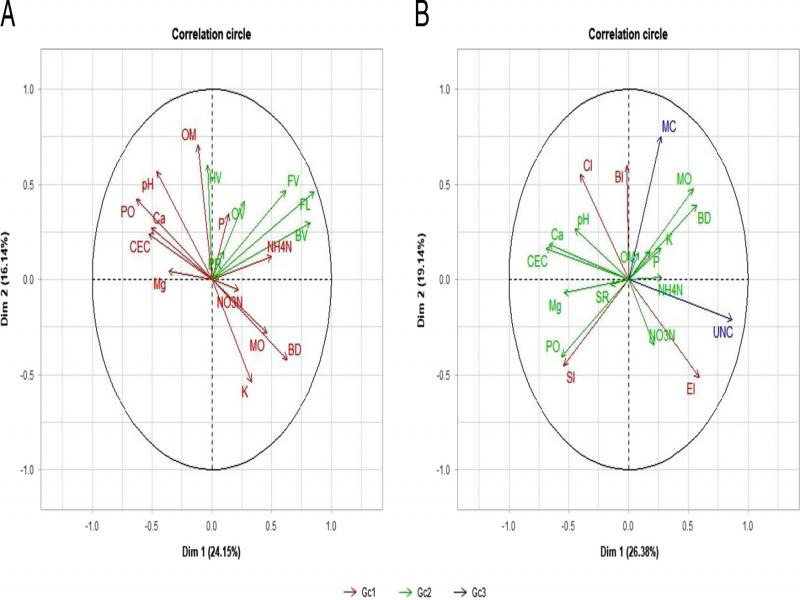
Multiple factor analysis of the variables where Dimension 1 (Dim 1) and Dimension 2 (Dim 2) represent the first and second best summary of variability of the information, respectively. (A) Relationships among soil properties (Gc1) (soil pH (pH), organic matter percentage (OM), nitrate-nitrogen (NO_3_-N), ammonium-nitrogen (NH_4_-N), calcium (Ca), magnesium (Mg), phosphorus (P), potassium (K), moisture percent (MO), bulk density (BD), cation exchange capacity (CEC), and porosity (PO) and abundance of nematode trophic groups (Gc2) (bacterivores (BV), fungivores (FV), omnivores (OV), predators (PR), herbivores (HV)) and non-herbivores (FL) (Yeates et al., 1993). (B) Relationships of soil food web indices (Gc1) (SI, EI, CI, and BI), soil properties (Gc2), and carrot yield and quality (Gc3) (total marketable (MC) and total unmarketable carrots (UNC)) from plots amended with integrated application of urea and plant compost.

Multiple factor analyses of nematodes, soil, and yield variables showed correlation patterns. Bacterivores and total non-herbivores were positively correlated with Dimension 1 while herbivores and organic matter content were positively correlated with Dimension 2 (Fig. 5A). Cation exchange capacity, calcium, and porosity were positively correlated to each other while negatively correlated with soil moisture content and bulk density. Potassium was negatively correlated with soil pH, organic matter, and herbivores. Bacterivores, fungivores, and non-herbivores were positively correlated with each other. As illustrated in Fig. 5B, cation exchange capacity and calcium were negatively correlated while total unmarketable carrot was positively correlated with Dimension 1. Total marketable carrot and BI were positively correlated with Dimension 2. CI was negatively correlated with EI. SI and porosity were positively correlated with each other, but negatively correlated with soil moisture content and bulk density.

**Figure 6:**
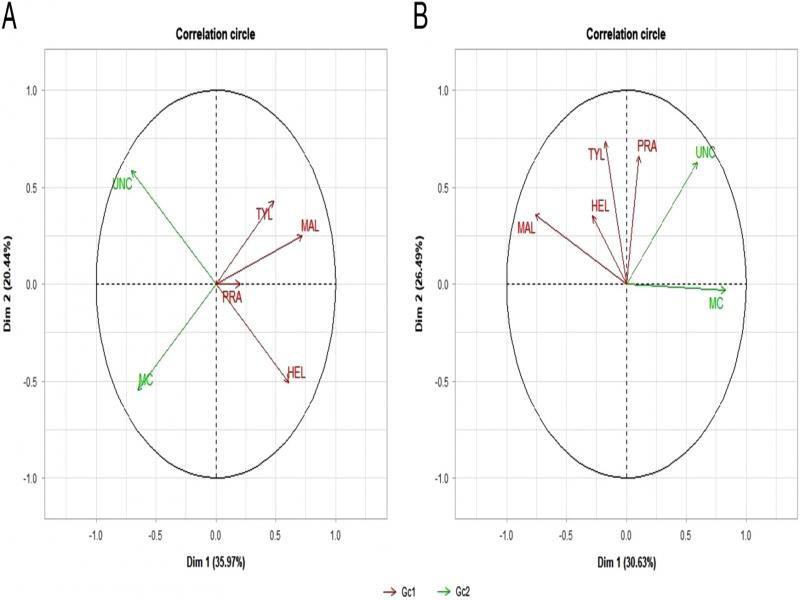
Multiple factor analysis of the variables where Dimension 1 (Dim 1) and Dimension 2 (Dim 2) represent the first and second best summary of variability of the information, respectively. (A) Relationships among abundant herbivores (Gc1) (*Malenchus*, MAL; *Tylenchus*, TYL; *Helicotylenchus*, HEL; *Pratylenchus*, PRA) and carrot yield and quality expressed as fresh weight (Gc2) (total marketable (MC) and total unmarketable carrots (UNC). (B) Relationships among and abundant herbivore nematodes (Gc1) and carrot yield and quality (Gc2) expressed as number from plots amended with integrated application of urea and plant compost.

Marketable carrot fresh weight was negatively correlated while *Tylenchus* and *Malenchus* (Fig. 6A). *Helicotylenchus* abundance was negatively correlated with total unmarketable carrot fresh weight. As illustrated in Figure 6B, the number of marketable carrots was positively correlated with Dimension 1. *Malenchus* was negatively correlated with the number of marketable carrots. *Tylenchus* and *Pratylenchus* were positively correlated with Dimension 2.

## Discussion

### Effect on nematode community structure

The expectation was that lower rates of urea mixed with higher rates of PC would improve nematode community structure by promoting nematodes from higher trophic group such as predators and omnivores in the soil food web. However, the result of the present study did not conform the expectation that increasing the rate of PC improves nematodes community structure. The assumption was that PC would improve soil physicochemical and biological properties conducive to the nematode community and reduce the toxic effect of urea on nematodes as well. Studies showed that integrated applications reduced the negative effect of inorganic fertilizers on soil organisms due to enhanced microbial activities and improved soil physiochemical properties (Pimentel et al., 2005; Evanylo et al., 2008; Lazcano et al., 2013). Integrated application was also regarded as a reasonable and effective approach to achieve both crop yields and sustainable agroecosystems by improving soil physicochemical and biological properties (Pimentel et al., 2005; Zhang et al., 2016).

The nematode trophic groups were not affected by treatments in the present study while previous works reported conflicting results. Inorganic nitrogen fertilization increased herbivores (Wang et al., 2006; Herren et al., 2020) and bacterivores (Song et al., 2016), but organic amendments are known to reduce herbivores (Melakeberhan et al., 2007; Mennan and Melakeberhan, 2010). In contrary, Hu and Qi (2010) reported compost increased bacterivores and herbivores compared with inorganic fertilizers, while Herren et al. (2020) reported compost has no effect on the nematode community. Generally, addition of compost with low C: N ratio, as in the present study, at least temporarily increased opportunistic bacterivores, probably due to high microbial activity (Bulluck et al., 2002a; Briar et al., 2011). Lack of significant increase in bacterivores and fungivores following treatments application in the present study could have been due to in adequate sampling intervals to detect possible short-term peaks in nematodes with short generation time. Bacterivore generation time, for example, is usually around 10 days after enrichment (Sánchez-Moreno et al., 2011). Variable experimental conditions, especially in field trails, might make generalization of the outcomes of different fertilization regimes difficult (Akhtar and Malik, 2000; Renčo et al., 2011).

### Effect on soil food web condition

The hypothesis was that lower rates of urea mixed with higher rates of PC would improve the soil food web condition by promoting nematodes of higher trophic group in the soil food web. Although none of the soil food web indices were significantly affected by the treatments, analyses of the faunal profile revealed that the soil food web structure was progressed overtime (Fig. 1). All treatments including the control had maturing and enriched soil food webs at harvest in 2014 while treatments with PC alone, and U1:PC1 significantly increased SI from 50. EI represents availability of nutrients in the soil to support the opportunistic nematodes (Ferris et al., 2001). Overall increase in omnivores over time attributed to the drastic increase in SI at harvest. Omnivores abundance was very low at planting because of farm activities such as tillage that adversely affect omnivores. The increase in omnivores at harvest was due to enough time for omnivores to reproduce (133 DAP) and other conducive environmental factors. Omnivore nematodes normally require 95 to 130 days to complete a life cycle (McSorley, 2012).

The significant increase in SI from 50 in the nematode faunal profile of the present study suggested that PC alone and U1:PC1 improved soil food web structure with greater trophic links (Ferris et al., 2012; Habteweld et al., 2018). SI values are usually low in agroecosystems because of physical and/or chemical disturbances of the soil (Fiscus and Neher, 2002; Berkelmans et al., 2003; Briar et al., 2011). The relatively greater structure in soil food webs in PC, and U1:PC1 may show reduced soil disturbances (Sanchez-Moreno et al., 2009; Zhang et al., 2016). Lack of significant increase in SI from 50 in U1:PC3 could be lack of the right proportion between urea and PC, and needs further investigation.

### Effect on soil physiochemical properties

Urea treatment significantly decreased soil pH while the other treatments increased soil pH overtime. After two years of the experiment, soil pH was low in urea plots compared with the other treatments except U3:PC1, which contain larger proportion of urea. Consistent to the present study, other studies showed that inorganic nitrogen fertilization decreased soil pH while compost amendment increased it (Bulluck et al., 2002b; Lie et al., 2010; Song et al., 2015; Han et al., 2016). Urea hydrolysis and subsequent nitrification result in release of hydrogen (H^+^) and may have led to a decline in soil pH in urea plots. Compost additions raise the pH of acid soils by forming aluminum complex and increasing base saturation (Shiralipour et al., 1992; Van den Berghe and Hue, 1999). Inorganic nitrogen fertilizers lower soil pH that, in turn, adversely affect soil biodiversity, overall soil health, and crop yield (Singh et al., 2013). However, increase in soil pH due to integrated application with higher rates of PC and PC alone is desirable, especially in acidic soils (Singh et al., 2013).

Urea application increased residual soil NO_3_-N at the end of the season compared with the rest of the treatments including non-amended control. Consistent with present study findings, Briar et al. (2007) found higher soil NO_3_-N in conventional system receiving inorganic fertilizers. In 2012, urea application resulted in significantly higher NO_3_-N at harvest where there were no plants in the field, suggesting that NO_3_-N could be lost to the environment through leaching. Surprisingly, most of the integrated applications had higher NO_3_-N at planting in 2013 compared with at planting and at harvest in 2012. This suggests residual effect of PC making nitrate available to the subsequent growing seasons through decomposition (Sánchez and Richard, 2009). Natsheh and Mousa (2014) found that in addition to yield increase, compost amendment increased soil fertility and reduced water requirement of the crop.

Urea, U3:PC1 decreased soil phosphorus content compared with the rest of the treatments except U1:PC1 (Fig. 2). This suggests that integrated applications supply other plant nutrients that inorganic fertilizers may not. In addition to delivering nutrients present in commercial fertilizers, compost includes nutrients that are sometimes not applied in adequate quantities by farmers (e.g. manganese, zinc, and sulfur). Thus, compost can serve as an insurance against potential yield limiting nutrients (Bulluck et al., 2002b). Moreover, the integrated application reduced the non-nutrient components (e.g. heavy metals) of composts such as biosolid compared with compost alone treatments that alleviate unintended consequences (Sikora and Knkiri, 2001).

The present study revealed that PC, and U1:PC3 and U1:PC1 significantly increased soil respiration compared with the rest of the treatments, indicating improved soil biological activity as we expected (Fig. 3). Ferris and Matute (2003) found improved soil respiration in treatments containing compost blended with wheat straw, but not from treatments with inorganic fertilizer and compost alone. Consistent with present study results, organic soil amendment increased rates of soil respiration compared with non-amended check (Gunapala et al., 1998; Treonis et al., 2010).

### Effect on carrot yield and quality

We expected greater quality of carrots from plots treated with integrated application due to readily available nitrogen fertilizer that support early growth, and improvement in soil physiochemical properties and pest suppression from PC (Abawi and Widmer, 2000; Lazcano et al., 2013; Aluko et al., 2014). In the present study, yield response was highly variable and difficult to make any conclusion. In previous studies, integrated application increased plant growth, yield, quality and soil fertility (Ahmed et al., 2006; Mahmoud et al., 2009). Keeling et al. (2003) also reported integrated application of nitrogen fertilizer with compost improved nitrogen utilization efficiency. Similarly, Anwar et al. (2005) reported integrated application of vermicompost and inorganic fertilizer performed the best with respect to growth, herb, dry matter, oil content, and yield of French basil. Content of principal constituents of basil oil were also higher under integrated nutrient management especially when vermicompost was applied with inorganic fertilizers (Anwar et al., 2005). Although we did not see yield increase in integrated application in this study, the benefits of enhanced biological activities and the anticipated reduction of negative environmental damage provide basis for further studies to test impact of integrated application on carrot yield and quality.

### Effect on correlations among nematode, soil, and yield parameters

Total herbivore nematode abundance was positively correlated with soil organic matter (Fig. 5A). Increase organic matter resulted in increased nutrient status and enhanced biological activity which promotes plant growth (Pimentel et al., 2005; Forge and Kempler, 2009). Enhanced plant growth probably increased the carrying capacity of plants on which herbivores feed (Bongers et al., 1997; Bongers and Bongers, 1998; Bongers and Ferris, 1999).

Positive correlation of bacterivores, total non-herbivores, and fungivores to ammonium nitrogen (NH4-N) would either show nutrient cycling ecosystem service provided by nematodes or enhancing effect of nitrogen fertilizer on bacterivore/fungivore nematodes (Bongers and Bongers, 1998). Both bacterivore and fungivore nematodes mineralize nitrogen in soil (Chen and Ferris, 1999; Ferris et al., 2004). Contributions of bacterivore nematodes are greater than those of fungivore nematodes (Ferris et al., 2004) and this was also reflected here with the degree of associations of bacterivores and fungivores to NH4-N. Nematodes contribute to nitrogen mineralization indirectly by grazing on decomposer microbes, excreting ammonium, and immobilizing nitrogen in live biomass. Nitrogen mineralization by nematodes may reach up to 8 to 19% of soluble nitrogen in soil (Neher, 2001). This is due to the fact that nematodes (C:N ratio of 8-12) have a lower nitrogen content than the bacteria (C:N ratio of 3-4) they consume (Wasilewska and Bienkowski, 1985).

Omnivores and predators positively correlated with soil phosphorus content in the present study. Such positive correlation would demonstrate that the raised pH and nutrient content in PC application enhanced the availability of food sources for omnivores and predators or created conducive environment for them. Consistent with the present study omnivores and predators were positively correlated to total phosphorus in plots with high manure application (Yang et al., 2016). Negative correlation of potassium with herbivores suggested suppressive effect on herbivore nematodes (Coyne et al., 2004).

One of the interesting correlations was that positive correlation between EI and NO3-N. Addition of nitrogen to the soil either due to fertilizer addition or mineralization by soil organisms increased enrichment opportunistic nematodes that would in turn increase EI, indicator of soil fertility (Ferris et al., 2001). That would imply the fertilizers used (Urea, PC) increased soil fertility.

The multiple factor analysis between the most abundant nematodes and carrot yield parameters showed correlation patterns. *Tylenchus* and *Malenchus* were negatively correlated with marketable carrot fresh weight while *Helicotylenchus* was negatively correlated with total unmarketable carrot fresh weight (Fig. 6A, B). However, considering that *Tylenchus* and *Malenchus* is a root hair feeder (Yeates et al., 1993; Bongers and Bongers, 1998), their negative correlation with carrot yield suggested the need for further investigation to avoid carrot damage from underestimated herbivore nematodes. The negative correlation between *Helicotylenchus* and unmarketable carrots was unexpected and difficult to give possible explanation.

In conclusion, the positive impact of U1:PC1 on SI suggested the potential positive impact of integrated applications on soil food web conditions. PC and integrated application mixed with higher rates of PC, increased soil pH, soil phosphorus content, and soil biological activity levels, while urea decreased soil pH and increased NO_3_-N. The negative correlation between *Tylenchus* and *Malenchus* with carrot yield indicated the need for further investigation to prevent potential yield loses from underestimated nematode groups. Although we did not see yield increase in integrated application in the present study, the benefits of enhanced biological activities, and increase in soil pH and phosphorus content together with the anticipated reduction in negative environmental damage would encourage further studies. One of the areas of the research could be mixing different compost types with inorganic fertilizers to exploit integrated application in carrot production systems.
